# The position of ground electrode affects electrocardiographic parameters in horses

**DOI:** 10.14202/vetworld.2022.1107-1112

**Published:** 2022-04-27

**Authors:** Wootichai Kenchaiwong, Pamika Sangpo, Anawach Kusol, Theerapong Pontaema, Wichaporn Lerdweeraphon

**Affiliations:** 1 Applied Animal Physiology Research Unit, Faculty of Veterinary Science, Mahasarakham University, Thailand; 2 Small Ruminant Research Unit, Faculty of Veterinary Science, Mahasarakham University, Thailand; 3 Network Center for Animal Breeding and Omics Research, Khon Kaen University, Thailand; 4 Faculty of Veterinary Science, Mahasarakham University, Thailand

**Keywords:** electrocardiogram, ground electrode, horses, position

## Abstract

**Background and Aim:**

Improper attachment of the grounding lead is one of the artifacts and causes difficulty in interpretation of ECG. This study aimed to examine the effects of the position of a ground electrode on electrocardiographic (ECG) parameters in horses.

**Materials and Methods:**

Sixteen Arabian horses without any cardiac problems were included in this study. The animals were divided into two groups, the base-apex lead method 1 (BA1 method) and the base-apex lead method 2 (BA2 method) with the reposition of the ground limb electrode to the xiphoid. ECG recordings (paper speed=25 mm/s and calibration=10 mm/mV) were performed to obtain six limb leads (leads I, II, III, aVR, aVL, and aVF). The amplitude and duration of P waves and QRS complexes, PR interval, QT interval, and T duration were analyzed. T wave morphology was observed. Heart rate was evaluated by using R-R interval in each recording.

**Results:**

Heart rate, P duration and amplitude, PR interval and T duration, and QRS duration and amplitude were not significantly different between the BA1 and the BA2 method, except that the BA2 method had a significantly higher QT interval than did the BA1 method (p<0.05). A higher significance of the percentage of coefficient of variation was seen on the P wave amplitude and the ORS amplitude in the BA1 method when compared to BA2 method (p<0.05).

**Conclusion:**

These data indicated that base-apex lead method with reposition of the ground limb electrode to the xiphoid can decrease variation of ECG configuration and might be useful for routine ECG evaluation and monitoring in horses. The limitation of this study was the evaluation of cardiac morphology and function by echocardiography to exclude cardiac problems. In further, the studies should examine the effect of breed, age, body weight, and sex on electrocardiography parameters in horses.

## Introduction

Exercise training has a majority effect on cardiovascular function, especially in horses with heart diseases, including atrioventricular insufficiency, ventricular septal defects, atrial fibrillation, and also supraventricular and ventricular arrhythmias [1,2]. Some of these abnormal rhythms can be detected by electrocardiographic (ECG) recordings. The ECG is the gold standard for diagnosis of cardiac arrhythmia [3] and is commonly used for the clinical examination of electrical disturbances in horses as a non-invasive and inexpensive tool [4-8]. Therefore, ECG is an important tool for assessing equine cardiac rate and rhythm in racing horses [9-11].

In horses, the base-apex lead system is frequently used for ECG recording, which is according to the principle of Einthoven’s triangle [6,12-14]. Due to the ventricular depolarization process in the horse being different from that of small animals, there is a widespread Purkinje network within the ventricles resulting in multiple depolarization points across the myocardium. Therefore, the base-apex lead method is limited in assessment of the frontal plane mean electrical axis (MEA) and has a consequent effect on calculations to detect cardiac chamber enlargement [15]. Many lead systems of recording the ECG have been developed to obtain an accurate assessment of the MEA and any rhythm for making a correct diagnosis in horses. The first, Dubois method, was described by Ayala *et al*. [16] and da Costa *et al*. [17]. The second, a modified precordial lead system, was described by some authors like Cherdchutham *et al*. [18]. Both of these methods repositioned the limb electrodes to the thorax. A recent study conducted by Hesselkilde *et al*. [12], a 12-lead system has given improvements by relocating the limb electrodes to the thorax to obtain a better ECG recording, called the Copenhagen method. The authors suggested that this method has numerous cables and electrodes, which can be complicated to approach with young or restless horses. Therefore, a base-apex lead method, where the three electrodes and one ground electrode may be more comfortable for the nervous horse. Although, whatever the method that is applied, it is important to allow rapid and improved variation in ECG recording.

ECG analysis is an important part of the diagnosis and assessment of any arrhythmias in horses [19]. Many artifacts on ECG recording may result in misdiagnosis and consequent inappropriate therapy. Therefore, it is important to recognize and eliminate these artifacts from the ECG recording. Improper attachment of the grounding lead is also one of the electrical interference artifacts that can cause difficulty in the interpretation of ECG [6]. However, the effect of the position of the ground electrode on ECG recording in horses has not been investigated. This study hypothesized that repositioning the ground limb electrodes (right hindlimb) to the xiphoid with the base-apex lead method might minimize variability of ECG measurement.

Therefore, this study aimed to examine the effects of the position of the ground electrode on ECG parameters in horses.

## Materials and Methods

### Ethical approval

All procedures performed on animals in this study were approved by Institutional Animal Ethics Committee, Mahasarakham University, Thailand (Approval number: IACUC-MSU-12/2021).

### Study period and location

This study was conducted from May to July 2021 at Faro Farm, Mueang District, Roi Et Province and SK Horse Stables, Mueang District, Sakon Nakhon Province, Thailand.

### Animals

A total of 16 Arabian horses were used for this study. The age of animals was 7.3±4.6 years with body weight of 394±48.8 kg. Clinical healthy animals were included in the study without any cardiac problems such as heart murmur and cardiac arrhythmias. The criteria of cardiac problems were based on results of auscultation and resting ECG recording.

### ECG examination

**Figure-1:**
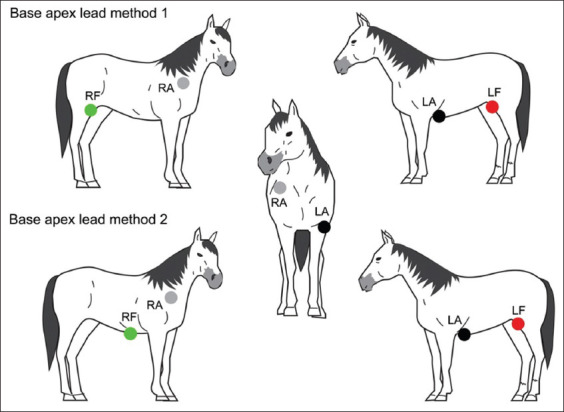
Electrode placement for the base-apex lead method 1 (top panel) and the base-apex lead method 2 (bottom panel).

**Figure-2:**
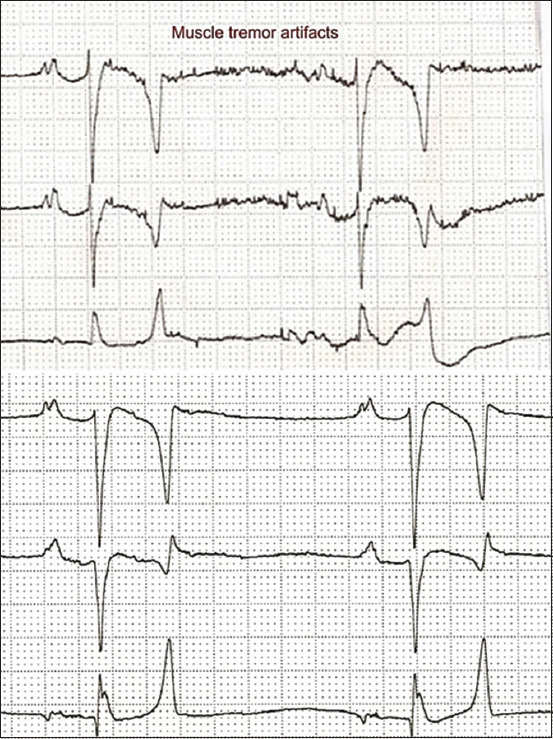
The presence of muscle tremor artifacts on electrocardiographic tracing (top panel) and the good quality of the electrocardiographic recording (bottom panel).

ECG recordings were performed on animals with a 3-channel electrocardiograph (Edan Instruments, Inc., VE-300, China) at a paper speed of 25 mm/s and calibration of 10 mm equal to 1 mV. Before recording, the animals were restrained in a standing position without any chemical restraint for acclimation within 5 min. All horses had ECG recordings in the base-apex lead systems with the different positions of the ground electrode. Recordings were made in the first method; the ground electrode (green electrode) was placed on the right stifle in the region of the patella (top panel in Figure-1), immediately followed by the second method, the ground electrode was relocated from the right stifle to the xiphoid process (bottom panel Figure-1). Four electrodes were placed on unshaved skin with alligator clips for all the standard bipolar limb leads (leads I, II, and III) and unipolar augmented limb leads (lead aVR, aVL, and aVF) in 1 min. The position of four electrodes in the two methods is shown in Table-1 and Figure-1. Lead I was selected to analyze the depolarization vector. The amplitude and duration of P waves and QRS complexes were measured together with PR interval, QT interval, and T duration. The amplitude of P wave was measured from the upper edge of the baseline to the peak of the P wave. Duration of P waves was measured at its inside, from the start to the end of the deflection from the baseline. P-R interval was measured from the beginning of the P wave to the beginning of the Q wave (R wave, if no Q wave was present). QRS duration was measured from the beginning of the first deflection to the end of the final deflection of the QRS complex. QRS amplitude was measured from the top edge of the baseline to the peak of the R wave. T duration was measured at its inside, from the start to the end of the deflection from the baseline. QT interval was measured from the onset of the QRS to the end of the T wave. P wave and QRS complex morphology were observed with consistent patterns in the ECG tracing. T wave morphology was observed with three patterns, including positive and negative deflection and biphasic. Heart rate was evaluated using R-R interval. All analyses were done by the same researcher. The main type of ECG artifact in this study was muscle tremor, shivering, and shifting stance of the horses. Muscle tremor is the high frequency (20-150 Hz) and/or medium frequency (3-5 Hz), causes very sharp and narrow multiple deflections of the baseline [20]. The presence of muscle tremor artifacts on ECG tracing is shown in Figure-2.

**Table 1:** Electrode placement.

Electrode	Base-apex lead method 1	Base-apex lead method 2
LA (black)	At 5^th^ intercostal space, just behind the point of the elbow of the left forelimb	Same position in method 1
RA (white)	At the right jugular furrow or in front of right scapula spine	Same position in method 1
LF (red)	On the loose skin at the left stifle in the region of the patella	Same position in method 1
RF (green or ground electrode)	On the loose skin at the right stifle in the region of the patella	On the skin at the xiphoid process

### Statistical analysis

All data were analyzed using an independent-t-test with SAS software University Edition (SAS, Inc., Cary, NC, USA) and p<0.05 was considered statistically significant. The variation of ECG parameters between the two methods was examined to obtain the percentage of coefficient of variation (%CV).

## Results and Discussion

**Figure-3:**
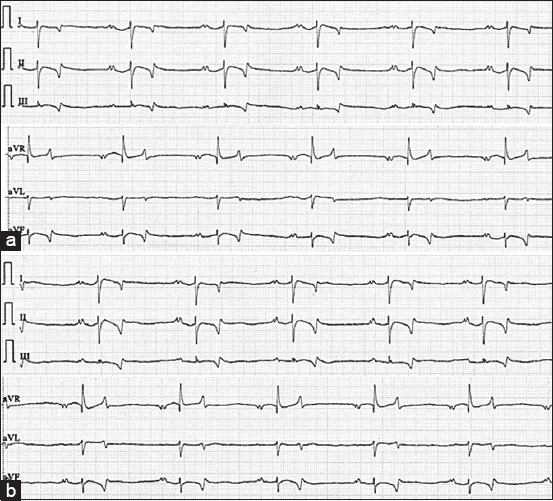
Example of a six-lead ECG for the base-apex lead method 1 (a) and the base-apex lead method 2 (b) from the same horse (paper speed=25 mm/s and sensitivity=10 mm/mV).

The ECG parameters were expressed as mean±standard deviation. Six-lead ECG recordings obtained with the BA1 and base-apex lead method 2 (BA2 methods) are presented in Figures-3a and b, respectively. The comparison of ECG parameters of horses between the two methods is shown in Table-2. The main causes of ECG artifacts in this study were muscle tremor, shivering, and shifting stance. These artifacts appear as a sequence of 5-150 Hz, which are frequently known to lead to sharp and narrow deflections of the baseline on ECG that make interpretation of the ECG difficult (Figure-2). There was a significant difference in QT interval between BA1 and BA2 methods (p<0.05). However, the other ECG parameters, including HR, P duration and amplitude, T duration, PR and QT interval, and QRS duration and amplitude were not significantly different. This study also found that T wave morphology was most likely expressed in biphasic configuration (50%), while 19% was found in positive configuration and 31% for negative configuration from 16 Arabian horses. The %CV of the base-apex lead method 1 (BA1 method) had significantly higher P wave amplitude and ORS amplitude than with the BA2 method (p<0.05), as shown in Table-3.

**Table 2:** The electrocardiographic parameters (mean±standard deviation) of horses between base-apex methods 1 and methods 2.

ECG parameters	Base-apex method 1	Base-apex method 2	p-value
HR (beats/min)	47.63±14.54	45.81±12.97	0.712
P wave duration (s)	0.14±0.04	0.13±0.04	0.365
P wave amplitude (mV)	0.27±0.13	0.20±0.06	0.071
PR interval (s)	0.30±0.05	0.28±0.04	0.126
QRS duration (s)	0.12±0.03	0.13±0.02	0.135
ORS amplitude (mV)	1.83±0.76	1.53±0.41	0.185
QT interval (s)	0.46±0.04	0.49±0.04	0.034
T wave duration (s)	0.14±0.03	0.14±0.03	1.000

**Table 3:** The percentage of %CV of ECG parameters between two methods.

ECG parameters	%CV		p-value of equality of variances

	Base-apex method 1	Base-apex method 2	
HR (beats/min)	30.52	28.31	0.6639
P wave duration (s)	29.96	28.32	0.5623
P wave amplitude (mV)	48.44	31.60	0.0082
PR interval (s)	18.05	15.82	0.389
QRS duration (s)	23.15	13.77	0.115
ORS amplitude (mV)	41.56	26.53	0.0209
QT interval (s)	9.01	8.59	0.9352
T wave duration (s)	20.86	23.36	0.6712

%CV=Coefficient of variation, ECG=Electrocardiographic

The main finding of this study was that ECG recording can eliminate artifacts such as muscle tremors by repositioning the ground limb electrode to the xiphoid process. This decreased variation in almost all ECG parameters with the base-apex method. The different ECG parameter values in this study are found to be in agreement with previous study conducted by Cherdchutham *et al*. [18]. We found that the repositioning of the ground electrodes did not affect the amplitude or duration of P waves and QRS complexes, T duration, and PR interval but did influence the QT interval. In addition, heart rate, the duration of ECG complexes, and intervals in the base-apex lead were within the normal reference range [15].

In our study, the configuration of the P wave and the ORS complex in the BA2 method were significantly more consistent than the BA1 method (p<0.05). This indicated that the repositioning of the ground electrode can reduce artifacts. It was possible that muscle tremors, shivering, and restlessness may be minimized with a ground electrode attached in an unobtrusive place such as the xiphoid position. The P wave was the dominant bifid configuration in the base-apex lead method, similar to previous studies [12,18]. The bifid P wave in horses revealed first the depolarization of the right atrium and subsequently the depolarization of the left atrium. We also found that the P wave can be a simple positive deflection (normal configuration some horses. The changing of heart rate may lead to changes in the P wave configuration. Thus, the varying P wave configurations are common in normal horses [6].

In this study, P, QRS, T duration, PR and QT interval were within the normal ranges, as reported by Cherdchutham *et al*. [18]. The QT interval was significantly lower in the BA1 method than the BA2 method (BA1=0.46±0.04 s and BA2=0.49±0.04 s). These alterations were most likely related to the muscle artifacts during ECG recordings. The ECG analysis incorrectly identified an earlier or later time for the start and end of ventricular depolarization and repolarization, respectively. Therefore, the accuracy of QT interval analysis depends on the corrective identification of the end of the T wave [21]. However, values of QT interval of both methods were no longer than 0.6 s, which was within the normal range [15].

In this study, the T wave was very variable in polarity and morphology in both methods. Several configurations of T wave may be due to the changing in heart rate. T wave may be helpful to differentiate between artifacts which can make the T wave disappear [22]. However, the T wave was the same or similar in an individual horse.

## Conclusion

This study indicated that a base-apex lead method with repositioning the ground limb electrode to the xiphoid can minimize the variation of ECG morphology, particularly the configuration of the P wave and the ORS complex. However, the positioning of the ground electrode did not affect the values of ECG parameters except QT interval. Thus, a modified base-apex lead method might be applied to the horses to screen cardiac arrhythmia. The cardiac morphology and function should be evaluated by echocardiography to exclude cardiac problems. In further studies, we can apply this knowledge to other horse breeds and should examine the effect of age, body weight, and sex on electrocardiography parameters for determining normal reference intervals in horses.

## Authors’ Contributions

WK: Designed the study and analyzed the data. PS, AK, TP, and WL: Recorded and analyzed the data. WL: Coordinated the study, drafted and revised the manuscript. All authors have read and approved the final manuscript.
